# Association between the *CEBPA* and *c-MYC* genes expression levels and acute myeloid leukemia pathogenesis and development

**DOI:** 10.1007/s12032-020-01436-z

**Published:** 2020-11-10

**Authors:** Adrian Krygier, Dagmara Szmajda-Krygier, Aleksandra Sałagacka-Kubiak, Krzysztof Jamroziak, Marta Żebrowska-Nawrocka, Ewa Balcerczak

**Affiliations:** 1 Laboratory of Molecular Diagnostics and Pharmacogenomics, Department of Pharmaceutical Biochemistry and Molecular Diagnostics, Medical University of Lodz, Muszynskiego 1 Street, 90-151 Lodz, Poland; 2 Department of Hematology, Institute of Hematology and Transfusion Medicine, Chocimska 5 Street, 00-791 Warsaw, Poland

**Keywords:** *CEBPA* gene, *c-MYC* gene, Transcription factors, Gene expression level, qPCR, AML, Adult leukemia

## Abstract

*CEBPA* and *c-MYC* genes belong to TF and play an essential role in hematologic malignancies development. Furthermore, these genes also co-regulate with *RUNX1* and lead to bone marrow differentiation and may contribute to the leukemic transformation. Understanding the function and full characteristics of selected genes in the group of patients with AML can be helpful in assessing prognosis, and their usefulness as prognostic factors can be revealed. The aim of the study was to evaluate *CEBPA* and *c-MYC* mRNA expression level and to seek their association with demographical and clinical features of AML patients such as: age, gender, FAB classification, mortality or leukemia cell karyotype. Obtained results were also correlated with the expression level of the *RUNX* gene family. To assess of relative gene expression level the qPCR method was used. The expression levels of *CEBPA* and *c-MYC* gene varied among patients. Neither *CEBPA* nor *c-MYC* expression levels differed significantly between women and men (p=0.8325 and p=0.1698, respectively). No statistically significant correlation between age at the time of diagnosis and expression of *CEBPA* (p=0.4314) or *c-MYC* (p=0.9524) was stated. There were no significant associations between relative *CEBPA* (p=0.4247) or *c-MYC* (p=0.4655) expression level and FAB subtype and mortality among the enrolled patients (p=0.5858 and p=0.8437, respectively). However, it was observed that *c-MYC* and *RUNX*1 expression levels were significantly positively correlated (rS=0.328, p=0.0411). Overall, AML pathogenesis involves a complex interaction among *CEBPA*, *c-MYC* and *RUNX* family genes.

## Introduction

Acute myeloid leukemia (AML) belongs to the group of heterogeneous neoplastic diseases of the white blood cell system. AML is characterized by clonal proliferation and growth of cancer-transformed blast cells that originate from the precursor myeloid cell in the bone marrow and in the peripheral blood [1, 2]. AML accounts for ~80% of all acute leukemias in adults and this number has been revealed to increase with age. AML most commonly occurs in older adults and is cured in approximately 35–40% of patients younger than the age of 60, however, in the group of patients >60 years, cases of full recovery are less common [1–4]. The course of acute myeloid leukemia is extremely severe: if left untreated, it can lead to the death of a patient within a few weeks. The pathogenesis of this disease is still not fully understood. Among genetic factors which can lead to AML development, chromosome aberrations (which are observed in 50–60% of AML patients) such as translocations: t(8; 21) (q22; q22) or (15; 17) (q22; q21), deletions: del (5q) or del (7q) and chromosomal inversions: inv (3), inv (8), or inv (16) can be distinguished [5]. The presence of mutations in the genes which are mainly responsible for proliferation and increasing the survival of progenitor cells, such as: *FLT3*, *RAS*, *KIT* or *TP53*, may also predispose to AML development [4]. Growing evidence point to the role of molecular indicators of tumor transformation, which may contribute to the formation of a self-regenerating leukemia cell clone. Among genetic aberrations potentially related to the development of AML or prognosis assessment among patients, changes in genes coding for the so-called transcription factors (including RUNX1, RUNX3, *CEBPA*, ASXL1) regulating transcription processes as well as controlling the cell differentiation and formation seem significant [4, 6].

Numerous studies support the complementary role of *CEBPA* and *c-MYC* transcription factors in risk stratification of hematologic malignancies development [7–12]. Additionally, according to the available data, the *RUNX1* gene influences and regulates the *CEBPA* and *c-MYC* gene expression [13, 14]. It has been proven that the deletion of the *RUNX1* gene reduces the mRNA level of the *CEBPA* gene. This leads to impaired bone marrow differentiation and may contribute to the leukemic transformation in cases of acute myeloid leukemia associated with the decreased RUNX1 activity [14]. On the other hand, properly functioning RUNX1 protein binds at three *c-MYC* distal enhancers where it represses *c-MYC* expression leading to apoptosis of AML cells [15].

CCAAT Enhancer Binding Protein Alpha (*CEBPA*) is an intronless gene located on chromosome 19q13.1. *CEBPA* gene encodes protein belonging to transcription factors family containing a basic leucine zipper (bZIP) domain which recognizes the CCAAT motif in the promoter regions of target genes [16]. C/EBPα protein regulates the expression of genes involved in cell cycle processes or homeostasis of body weight [16, 17]. Furthermore, C/EBPα is a critical regulator of granulopoiesis and its expression enables hematopoietic progenitors to differentiate [18]. Growing evidence indicate that *CEBPA* gene probably acts as a tumor suppressor in hematologic and non-hematologic malignancies [19]. Moreover, mutations of this gene are associated with acute myeloid leukemia.

Another gene belonging to the transcription factors is BHLH Transcription Factor *c-MYC* gene. *c-MYC* is a proto-oncogene which is located on chromosome 8 q24.21. It encodes a nuclear phosphoprotein that plays an essential role in cell cycle progression, cellular transformation and apoptosis [20, 21]. Furthermore, encoded protein forms a complex with the related transcription factor MAX, which binds with the E box DNA consensus sequence and regulates specific target gene transcription. *c-MYC* is associated with Burkitt Lymphoma or High-Grade B-Cell Lymphoma development [22, 23]. Recent studies indicate that the expression of proto-oncogene MYC is also tightly regulated during hematopoiesis [24]. Various studies suggest that *c-MYC* gene is dysregulated in cancers, including leukemias. The expression of *c-MYC* is highest in hematopoietic stem cells (HSCs) and decreases during myeloid differentiation [11]. There is also an association between the activity of *c-MYC* and *CEBPA* genes. Downregulation of MYC expression causes repression on key target genes, such as *CEBPA* or *GADD45A* and, in consequence, it initiates hematopoietic differentiation and apoptosis, respectively [25].

Understanding the function and full characteristics of *CEBPA* and *c-MYC* genes in the group of patients with acute myeloid leukemia can be particularly helpful in assessing prognosis, and their usefulness as prognostic factors can be revealed. This may translate into the development of new targeted therapeutic strategies and an increase in the effectiveness of treatment in patients with acute myeloid leukemia.

The aim of the study was to evaluate *CEBPA* and *c-MYC* mRNA expression level and their association with clinical and pathological features of AML patients. Moreover, the obtained results of selected genes expression levels were correlated with the expression level of genes belonging to the *RUNX* family (including *RUNX1* and *RUNX3*) evaluated for the same study group (published data).

## Materials and methods

### Study group

The study population comprised 46 patients (22 females and 24 males). All recruited patients were diagnosed with AML at the Hematology Clinic, the Medical University of Lodz (Lodz, Poland) and the Institute of Hematology and Blood Transfusion (Warsaw, Poland). The median age at the time of AML diagnosis was 61.5 years (17–80 years). The Ethics Committee of Medical University of Lodz approved the present study (protocol number RNN/88/16/KE) and it was in accordance with the principles of the Declaration of Helsinki. Written informed consent was obtained from the all recruited patients. All data collected in the study were anonymous. All demographic and clinical characteristics of patients are presented in Table 1.

**Table 1:** Demographic and clinical characteristics of patients

Characteristics	Number of patients (n=46)
Gender	
Men	24
Women	22
Age at the time of AML diagnosis (years)	
Range (mean)	17-80 (61.5)
Leukemia subtype according to FAB classification (%)	
M0	2.2 (n=1)
M1	8.7 (n=4)
M2	17.4 (n=8)
M3	4.3 (n=2)
M4	13.0 (n=6)
M5	6.5 (n=3)
M6	2.2 (n=1)
Undefined AML	45.7 (n=21)
Mortality (%)	43.5 (n=20)
Leukemia cells karyotype (%)	67.4 (n=31)
Normal	41.9 (n=13)
Abnormal	58.1 (n=18)

*n-number of cases*

### Material

The investigated material comprised peripheral blood samples which were obtained during routine blood tests.

### RNA isolation and cDNA synthesis

The total RNA was isolated from peripheral blood samples using the Total RNA Mini Kit (A&A Biotechnology, Gdynia, Poland) according to the protocol. The A260/280 ratio was determined to assess the purity of extracted RNA. The obtained RNA required for reverse transcription was pure (the range of A260/280 ratio was between 1.6 and 1.8). The obtained RNA samples were stored at -80°C until further analysis. In order to obtain cDNA from RNA, the reverse transcription reaction was performed according to the manufacturer's protocol using a High Capacity cDNA Reverse Transcription kit (Applied Biosystems; Thermo Fisher Scientific, Inc., Waltham, MA, USA). The final concentration of RNA in the reaction mixture in samples was equated to 0.02 μg/μl. The reaction parameters were following: 25 °C for 10 min, 37 °C for 120 min and 85 °C for 5 min.

### Polymerase chain reaction (PCR)

The PCR was carried out for qualitative analysis of *CEPBA* and *c-MYC* expression according to the manufacturer's protocol for the AccuTaq™LA DNA Polymerase kit (Sigma Aldrich; Merck KGaA, Darmstadt, Germany). The reaction mixture contained: 0.7 μl of 10 μM of each primer (*CEBPA* and *c-MYC* respectively), 3.5 μl of 1.5 mM 10x PCR buffer without MgCl_2_ (Sigma Aldrich; Merck KgaA), 0.7 μl of 25 mM MgCl_2_, 0.4 μl of 0.2 mM dNTP (deoxynucleotides) mix, 0.2 μl of 0.5 U AccuTaq LA DNA Polymerase, 1 μl of cDNA and 13.8 μl distilled water. The final volume of reaction mixture was 21 μl. For each experiment a negative control without cDNA template was included. The PCR amplifications for investigated and reference genes were carried out using an MJ Mini Personal Thermal Cycler (BioRad Laboratories, Inc., Hercules, CA, USA). Thermal conditions were as follows: initial denaturation at 95°C for 2 min, denaturation at 95°C for 1 min, annealing at 56°C for *CEBPA* and 57°C for *MYC* for 30 sec, elongation at 72°C for 45 sec and final elongation at 72°C for 5 min. For visualization of PCR product, electrophoresis on a 2% agarose gels were used.

### Real-time PCR

In order to assess *CEBPA* and *c-MYC* mRNA expression level, qPCR analysis was performed using RotorGene-™ 6000 thermocycler (Corbett Life Science; Qiagen GmbH, Hilden, Germany) for *CEBPA* and Stratagene Mx3000P (Agilent Technologies, Germany) for *c-MYC*. Experiments for investigated and reference genes (*GAPDH*) were performed in triplicates. For *CEBPA* gene, the reaction tube consisted of 5 μl RT HS-PCR Mix Sybr® B (A&A Biotechnology, Gdynia, Poland), 0.7 μl of 10 μM of each primer, 2.6 μl of nuclease-free water and 1 μl of cDNA template. The final volume of each tube was 10 μl. For *c-MYC* gene, the reaction mixture consisted of 7.5 μl JumpStart Taq ReadyMix (Sigma Aldrich, Germany), 0.5 μl of 10 μM of each primer, 0.2 μl of ROX Reference Dye 2, 6.3 μl of nuclease-free water and 1 μl of cDNA template. The final volume of each tube was 16 μl. Thermal cycling parameters for *CEBPA* gene were as follows: initial denaturation at 95°C for 10 min, denaturation at 95°C for 10 sec, primer annealing at 56°C for 15 sec, elongation at 72°C for 20 sec. The reaction conditions for *c-MYC* gene were as follows: initial denaturation at 95°C for 10 min, denaturation at 95°C for 45 sec, primer annealing at 56°C for 45 sec, elongation at 72°C for 45 sec. In every experiment a negative control without cDNA template was included. The analysis of melting curves was performed to assess the specification of products. The 2^–ΔΔCq^ method was used to estimate relative changes in gene expression determined by RTqPCR- analysis.

### Statistical analysis

Statistical analysis was conducted using STATISTICA 13.3 (StatSoft Inc., Tulsa, OK, USA). Conformity with the normal distribution was checked using Shapiro-Wilk test. A comparative statistical analysis was performed using Student’s t-test, MannWhitney U test, Kruskal–Wallis one-way analysis of variance and Spearman's rank correlation coefficient. P-value <0.05 was considered as significant in all conducted tests.

## Results

### Relative *CEBPA* and *c-MYC* expression level in the study group

In all 46 samples *GAPDH* expression was detected. The presence of *c-MYC* was identified in 43 samples and presence of *CEPBA* expression was detected in 44 samples. In the study group the expression levels varied, ranging between 0.01 and 3.94, with a median value of 1.57 for the *CEBPA* gene and between 0.05 and 2.07, with a median value of 0.49 for the *c-MYC* gene.

### Connection between *CEBPA* and *c-MYC* expression level and gender and age of diagnosis

The study group comprised 22 females (47.8%) and 24 males (52.2%). Neither *CEBPA* nor *c-MYC* expression levels differed significantly between women and men (p=0.8325 and p=0.1698, respectively).

The median age at the time of AML diagnosis was 61.5 years (min. 17.0, max. 80.0). No statistically significant correlation between age at the time of diagnosis and expression of *CEBPA* (p=0.4314) or *c-MYC* (p=0.9524) was stated.

### Relative *CEBPA* and *c-MYC* gene expression level according to FAB classification, mortality and leukemia cell karyotype

**Fig. 1:**
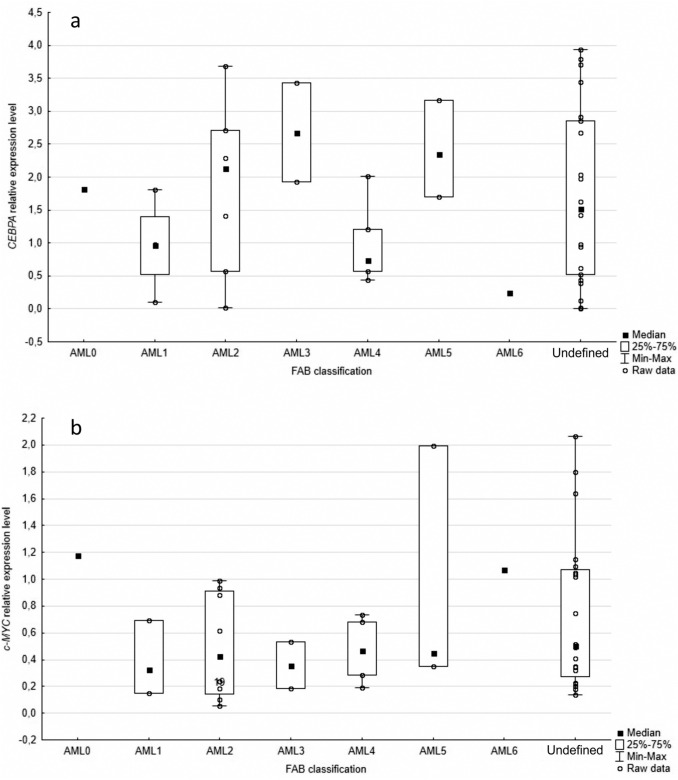
Association between *CEBPA* (a) and *c-MYC* (b) genes expression levels and FAB classification, no significant differences were observed (p=0.4247; p=0.4655, respectively)

Patients were divided into subgroups according to the FAB classification of AML (M0 2.2%, M1 8.7%, M2 17.4%, M3 4.3%, M4 13.0%, M5 6.5%, M6 2.2%, undefined 45.7%). The median *CEBPA* expression level was the highest in the M3 and the lowest in M6. The median *c-MYC* expression level was the highest in M0 and the lowest in M6. The differential expression of *CEBPA* and *c-MYC* between the different FAB subtypes is shown in Fig. 1. There were no significant associations between relative *CEBPA* (p=0.4247) or *c-MYC* (p=0.4655) expression level and FAB subtypes.

Next, association between genes expression levels and mortality among the enrolled patients was analyzed. However, no statistically significant difference was found (*CEBPA* p=0.5858, *c-MYC* p=0.8437).

Leukemia cell karyotype was determined in 67.4% of enrolled patients, among them abnormal karyotype was found in 58.1% of cases. Subgroups of patients with abnormal and normal karyotype of leukemia cells did not differ significantly in neither *CEBPA* (p=0.5500) nor *c-MYC* (p=0.6370) expression level.

### Interrelation between *CEBPA*, *c-MYC*, *RUNX1* and *RUNX3* expression level

**Fig. 2:**
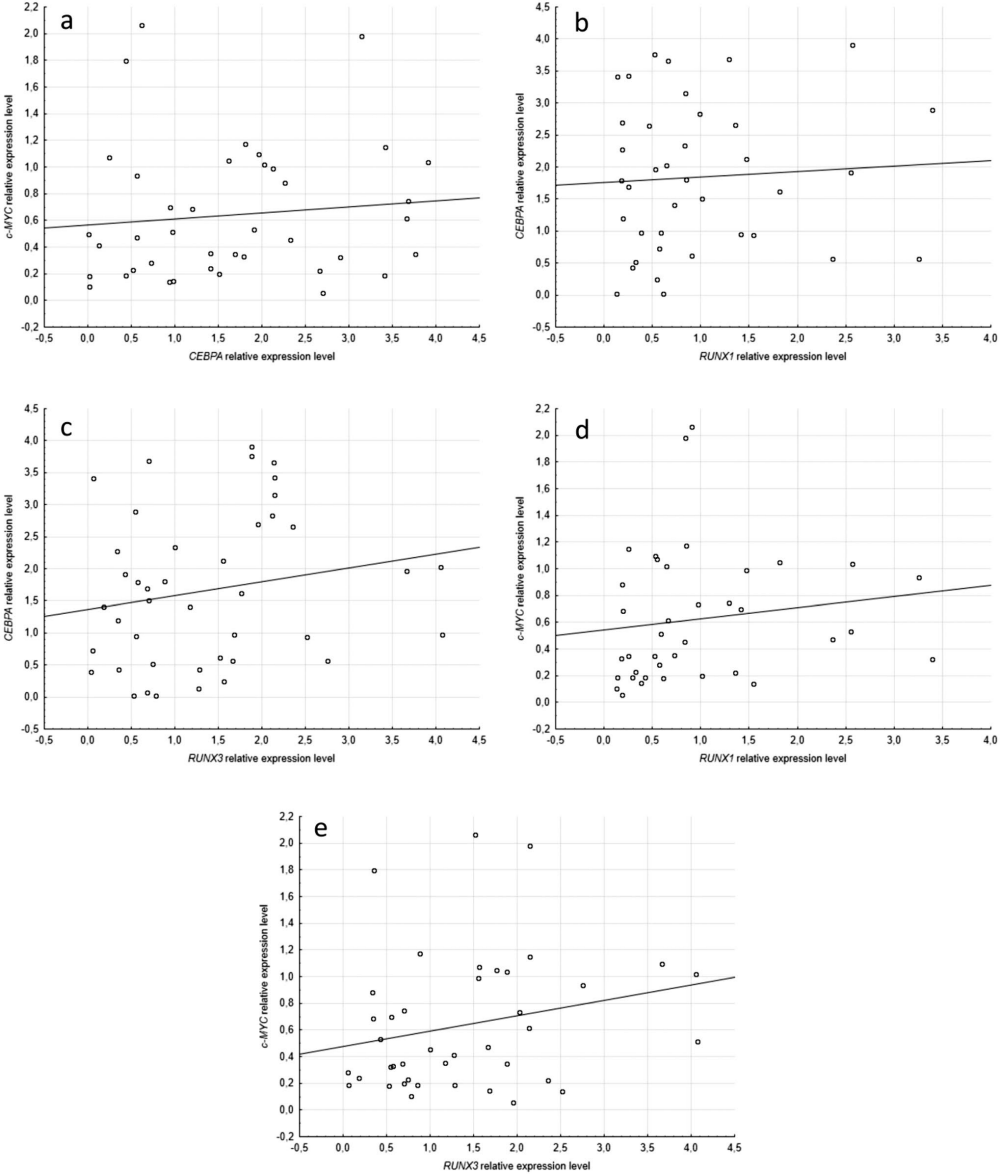
Interrelation between *CEBPA*, *c-MYC*, *RUNX1*, *RUNX3* expression levels. *CEBPA* and *c-MYC* gene expression levels were not significantly related (r_S_=0.203, p=0.2039, **a**). *CEBPA* expression level was not significantly associated with either *RUNX1* or *RUNX3* expression level (r_S_=0.013, p=0.9366 and r_S_=0.270, p=0.0875, **b** and **c**, respectively). *c-MYC* and *RUNX1* expression levels were significantly positively correlated (r_S_=0.328, p=0.0411, **d**). *c-MYC* and *RUNX3* expression levels was not statistically significantly connected (r_S_=0.256, p=0.1061, **e**)

Additionally, interrelation between expression levels of *CEBPA* and *c-MYC* genes was evaluated. We found no connection between expression levels of analyzed genes (r_S_=0.203, p=0.2039, Fig. 2a). Moreover, using previously published data [26] in the same patient cohort, the connection between expression levels of *CEBPA* and *c-MYC* and *RUNX1* and *RUNX3* expression level was analyzed. *CEBPA* expression level was not significantly associated with either *RUNX1* or *RUNX3* expression level (r_S_=0.270, p=0.0875 and r_S_=0,013, p=0.9366, Fig. 2b, c, respectively). Contrarily, *c-MYC* and *RUNX1* expression levels were significantly positively correlated (r_S_=0.328, p=0.0411, Fig. 2d). *c-MYC* and *RUNX3* expression levels was not statistically significantly connected (r_S_=0.256, p=0.1061, Fig. 2e) in the analyzed patient cohort.

## Discussion

In the studied AML cohort, we stated the ubiquitous expression of *CEBPA* and *c-MYC,* the level of the expression varied substantially between cases. In previous research *CEBPA* overexpression was shown in vast percentage of AML cases [27, 28]*,* but a counter-observation was also published [29]*.* Also, in vast majority of AML patients *c-MYC* overexpression was shown [30]*.* In the present paper, no association was found between the expression of genes and neither gender nor age at the time of diagnosis, which matches the observations made earlier [30–32]*.* Gholami et al. found a significant upregulation of *CEBPA* in male AML patients [33].

The expression of C/EBPα initiates with the commitment of multipotential precursors to the myeloid lineage. It is specifically upregulated during a granulocytic differentiation, but downregulated during the monocytic pathway [34, 35]. Downregulation of *CEBPA* expression at the transcription, translation or post-translation level could inhibit the myeloid differentiation and, simultaneously, trigger the cell cycle progression. D’Alò et al. demonstrated that *CEBPA* expression was significantly lower in mature granulocytes and monocytes in comparison to bone marrow mononuclear cells, including hematopoietic progenitor cells. AML cases positive of the myeloid differentiation markers CD33 and CD11c had higher levels of *CEBPA* [36]. Considering data mentioned above and the degree of maturity of leukemia cells that is the basis of the French-American-British (FAB) AML classification system, we sought for connection between *CEBPA* expression level and FAB subtypes.

In studied cohort *CEBPA* expression level varied between FAB subtypes of AML. Heterogeneous *CEBPA* expression among AML subtypes was also reported in previous studies [34, 36]. Recently, Gholami et al. have shown that *CEBPA* is significantly overexpressed in M0, M3 and M4 FAB subtypes [33]. In the present paper, the highest expression of the gene is in the M3 subgroup. This stays in agreement with the results obtained by others [18, 34] which found that acute promyelocytic leukemia t (15;17) cases are characterized by overexpression of *CEBPA*. A similar observation was made by Kassem et al. [29] but some contrary results were also reported [27] (Salarpour et al., 2017)*.* D’Alo et al. showed the lowest *CEBPA* levels in acute erythroid leukemia in relation to other studied AML subtypes [36]. Also, in the present research, a low *CEBPA* expression in the studied cohort was stated for the M6 case. However, a statistical significance of the difference in the *CEBPA* expression level between AML subtypes was confirmed probably neither in the present research nor by others [27, 36], due to a small number of patients.

Also, the differences in the *c-MYC* expression level between FAB subtypes was observed in the study cohort. It stays in agreement with the previous conclusion made by Ohanian et al. [30] - mean MYC-immunopositivity in AML WHO subtypes differ from 6% to 55.8% in various AML WHO subtypes. Interestingly, they found a low *c-MYC* expression level in acute monoblastic/monocytic leukemia, whereas in our study a relatively high *c-MYC* expression was often noted in the M5 subtype.

The role of *CEBPA* and *c-MYC* pathogenesis of myelodysplastic diseases is essential but also the influence of the genes on outcome of AML patients is still an unresolved clinical question. In research published by Kassem et al., a higher *CEBPA* expression correlated with a higher overall survival in AML compared to a low *CEBPA* expression [29]***.*** Other investigators reported [28] that no significant difference in event-free and overall survival among AML patients with different *CEBPA* expression levels were apparent. Similarly, no association with EFS and OS over the period of 3 years was stated by Grossmann et al. [18]. Also, in the present study, *CEBPA* expression level was not associated with mortality of patients.

Falantes et al. observed a high *c-MYC* expression level and that was connected with a shorter survival in the univariate but not multivariate analysis in MDS in AML cases [31]. In the present research, *c-MYC* expression level was not associated with mortality, but it could be assumed that the result may be cohort-dependent. Yun et al. previously proved that a high *c-MYC* expression is an independent poor prognostic factor for the overall survival outcome in AML, however, the influence on survival was confirmed only in cases of no *TP53* somatic mutations or del(17p) [32]. Moreover, MYC protein influenced negatively overall survival in favorable and an intermediate cytogenetic risk group, but it has a protective effect in the unfavorable risk group [37]. In the present publication, results from a karyotype analysis were available for a few cases only.

A karyotype analysis is one of the basic tools to stratify AML patients into different risk groups. In the present paper, the association between *CEBPA* expression level and blast cell karyotype was not found. This is in contradiction with the previously published results, where the *CEBPA* expression was significantly increased in a favorable and adverse cytogenetic risk group and in AML patients with abnormal karyotype [33]. Moreover, it was shown that in AML of intermediate risk, the karyotype *CEBPA* low expression level seems to be associated with a poor prognosis for patients [28].

There is some evidence that *c-MYC* has a growth-inducing role and can be connected with MDS progression in AML. A study published by Poloni et at. revealed that MDS patients with a favorable karyotype had levels of *c-MYC* significantly lower than patients with an intermediate and unfavorable karyotype [38]. It has also been shown that *c-MYC* expression increased from relatively low in healthy control and low-risk MDS, through intermediate in high-risk MDS, to high in AML patients [31]. In the same study, *c-MYC* expression was not associated with either MDS or AML karyotype. It stays in agreement with our results, where *c-MYC* expression level did not differ between AML patients with normal and aberrant karyotype. Interestingly, the highest expression of MYC protein was noted in AML patients with a favorable cytogenetic risk group [37].

A switch between proliferation and differentiation in early myeloid precursor cells is a key step during granulopoiesis and it is reciprocally controlled by C/EBPα and *c-MYC*. It was shown that Max, a heterodimeric partner of *c-MYC*, is one of the interacting proteins of C/EBPα in myeloid U937 cells and acts as coactivator [39]. Moreover, C/EBPα can directly downregulate a human *c-MYC* promoter activity and expression level, and thus induce cellular differentiation in myeloid cell line [40]. On the other hand, C/EBPα protein level is repressed in stable cell lines overexpressing Myc [41]. Gene expression profiling revealed that *CEBPA* and *c-MYC* genes are among the most overexpressed genes in AML [42, 43]. Taking this into account, we sought the connection between *CEBPA* and *c-MYC* expression levels in the studied AML cohort, however we did not find any correlation between the levels.

Several studies indicated that *CEBPA* and *c-MYC* could be interrelated with *RUNX1*, an essential transcription factor in leukemogenesis. In the Jurkat human and murine T cell line, primary hematopoietic Runx1 was shown to repress *c-MYC* transcription in a C-terminal- and DNA-binding-dependent manner [15]. It binds at three MYC distal enhancers, where it represses MYC expression leading to apoptosis of AML cells [44]. Recently, Weng et al. demonstrated that GM-CSF attenuates MYC-associated gene signatures in t (8;21) (RUNX1-ETO) leukemia cells, but not in control cells by restoring the expression of a subset of MYC-repressed targets (e.g. *CEBPA)*, which promote a myeloid differentiation and apoptosis [26]. MYC and RUNX1 with two other factors, SP1 and GATA2, form multi-protein transcription complex which activates expression of *SET*, encoding important oncoprotein for AML development. MYC increases the expression of the other three transcription factors of the complex, and supports their recruitment to the promoter of *SET* [45]***.*** In the present publication, the significant positive correlation between *c-MYC* and *RUNX1* expression levels was found, which could suggest a different or more complex reciprocal regulation of the genes in vivo. Simultaneously, *c-MYC* and *RUNX3* expression levels were independent.

Grossmann et al. showed that among AML cases with normal karyotype *RUNX1*-mutated cases have a lower *CEBPA* expression than wild-type cases. *CEBPA* expression was also lower in AML t(8;21)/*RUNX1-RUNX1T1* cases than in AML cases with other karyotypes [18]. Thus, downregulation of *CEBPA* may contribute to leukemogenesis in *RUNX1*-mutated AML. It stays in agreement with observation of Salarpour et al. which previously demonstrated that *CEBPA* and *RUNX1* expression levels are significantly positively correlated in both AML patients and healthy volunteers, although correlation was stronger in normal control cases [27]. Researchers suggested that the regulatory network between these genes could be disrupted in AML. In the present publication, no correlation between *CEBPA* and neither *RUNX1* nor *RUNX3* expression levels was found, which supports this hypothesis.

It should be noted that the undertaken study has some limitations. The presented results are limited to the Polish population with restricted sample size. In order to confirm the outcomes, further investigations on larger AML patients' cohorts from diverse populations are necessary. Lack of association between selected demographic and clinical characteristics may be caused by a relatively small study group. Future trials would benefit from increasing the number of subjects. Overall, AML pathogenesis involves a complex interaction among *CEBPA*, *c-MYC* and *RUNX* family genes.

## Data Availability

The datasets used and/or analyzed during the current study are available from the corresponding author on reasonable request.
